# MicrobiomeKG: bridging microbiome research and host health through knowledge graphs

**DOI:** 10.3389/fsysb.2025.1544432

**Published:** 2025-08-29

**Authors:** Skye L. Goetz, Amy K. Glen, Gwênlyn Glusman

**Affiliations:** ^1^ Institute for Systems Biology, Seattle, WA, United States; ^2^ School of Electrical Engineering and Computer Science, Oregon State University, Corvallis, OR, United States

**Keywords:** systems biology, data integration, supplementary data, table mining, knowledge representation, health informatics, hypothesis generation, neural networks

## Abstract

The microbiome represents a complex community of trillions of microorganisms residing in various body parts and plays critical roles in maintaining host health and wellbeing. Understanding the interactions between microbiota and their host offers valuable insights into potential strategies for promoting health, including microbiome-targeted interventions. We have created MicrobiomeKG, a knowledge graph for microbiome research, that bridges various taxa and microbial pathways with host health. This novel knowledge graph derives algorithmically generated knowledge assertions from the supplementary tables that support published microbiome papers. By identifying knowledge assertions from supplementary tables and expressing them as knowledge graphs, we are casting this valuable content into a format that is ideal for hypothesis generation. To address the high heterogeneity of study contexts, methodologies, and reporting standards, we leveraged neural networks to implement a standardized edge scoring system, which we use to perform centrality analyses. We present three example use cases: linking helminth infections with non-alcoholic fatty-liver disease via microbial taxa, exploring connections between the *Alistipes* genus and inflammation, and identifying the *Bifidobacterium* genus as the most central connection with attention deficit hyperactivity disorder. MicrobiomeKG is deployed for integrative analysis and hypothesis generation, both programmatically and via the Biomedical Data Translator ecosystem. By bridging data gaps and facilitating the discovery of new biological relationships, MicrobiomeKG will help advance personalized medicine through a deeper understanding of the microbial contributions to human health and disease mechanisms.

## 1 Introduction

The microbiome represents a complex community of trillions of microorganisms that reside in various body parts; it plays critical roles in maintaining host health and wellbeing. Emerging research has revealed that it influences numerous physiological processes, including digestion (Hills et al., 2019), aging (Wilmanski et al., 2021), and immune system function (Wiertsema et al., 2021). Conversely, the dysregulation of microbiota (dysbiosis) is associated with various diseases and negative health outcomes (e.g., inflammatory bowel disease, obesity, diabetes, and neurological disorders) (Hills et al., 2019). Hence, understanding the interactions between microbiota and their host offers valuable insights into potential strategies for promoting health, including microbiome-targeted interventions.

The NCATS Biomedical Data Translator (“Translator”) is a cutting-edge platform that aims to revolutionize biomedical research (Fecho et al., 2025). It integrates vast amounts of diverse data, from genes to clinical records, and uses advanced algorithms to uncover insights and accelerate discoveries. By harmonizing data and enabling semantic searches, Translator fosters a collaboration among researchers and facilitates the development of new treatments and therapies for various diseases. The Translator project uses knowledge graphs (KGs) to store the wealth of data required for reasoning in a compact, easy-to-parse, universal format. KGs organize data from multiple sources, capture information about entities of interest in a given domain or task, and display connections between them. KGs comprise nodes (things) and edges (relationships between things).

Some prominent projects have come close to reconciling microbiome data with Translator philosophy. BugSigDB (Geistlinger et al., 2024) serves as a comprehensive database of published microbial signatures but lacks content connecting the microbiome and host health, as well as a knowledge graph format. KG-Microbe (Santangelo et al., 2025) is an integratively analyzable knowledge graph linking prokaryotic data for phenotypic traits, taxonomy, chemicals, and environment descriptors, but is yet to include content linking the microbiome and host health. MicroPhenoDB (Yao et al., 2020) incorporates content linking the microbiome with host health but lacks a knowledge graph format. MetagenomicsKG (Ma et al., 2024) incorporates multiple content sources, inclusive of microbiome-host health knowledge, into an integratively analyzable knowledge graph. However, neither includes knowledge from supplementary tables in their findings.

Here, we present MicrobiomeKG, an integratively analyzable knowledge graph for microbiome research that bridges various taxa and microbial pathways with host health, built from algorithmically generated knowledge assertions from supplementary tables and deployed to Translator (Fecho et al., 2025).

## 2 Methods

### 2.1 Selection of publications and supplementary tables

The publications included in the initial version of MicrobiomeKG represent a manually selected, non-comprehensive set of recent and multiomic-driven scientific papers that (a) bridge microbiome and host health-related content and (b) include one or more supplementary tables with content that can be modeled as subject–predicate–object triples (e.g., taxon X affects disease Y) —the standard units of knowledge graphs.

### 2.2 Derivation of knowledge assertions

Leveraging relevant content from the supplementary tables, their descriptions, or the manuscript itself, we incorporated supplementary data contents into DataFrames using Python’s “polars” library and processed the content to derive assertions. We implemented a declarative data transformation system that paired a human-curated configuration file to each supplementary table; the configuration file specified the transformations required to extract the knowledge assertions. We used custom Python scripts to transform the DataFrames values in multiple ways via operations on individual values and on entire rows. Value transformations included mathematical transformations (e.g., exponentiating log-transformed p-values), extracting relevant content with regular expressions (e.g., extracting “Actinobacteria” from “kurilshikov_class.Actinobacteria.id.419”), and text cleaning (e.g., deriving “enterocloster bolteae” from “enterocloster_bolteae”). Row operations included filtering based on certain conditions (e.g., based on a given column’s Boolean value), dropping duplicates, dropping null values, and imposing cutoffs for filtering. Some edge attributes were manually computed when not provided but were reasonably inferred (e.g., total cohort size for meta-analyses where the cohort sizes for all initial analyses are made explicit). Such manual operations were performed only in the creation of the configuration files but not in post-processing the extracted knowledge assertions; this step was entirely automated and objective. We use a p-value cutoff of 0.1 so that the graph contained both statistically significant and not significant but highly suggestive edges.

### 2.3 Standardization of KG contents and structure

We standardized all edge predicates and node categories to Biolink ontology predicates and Biolink ontology classes (Unni et al., 2022). Furthermore, we mapped nodes to ontologies, representing them using compact universal resource identifiers (CURIEs) and normalizing them using BABEL (version of 2025/03/31)^1^. We dropped any knowledge assertions that failed to map subject or object to standard CURIEs. We then exported the output in Knowledge Graph Exchange (KGX) tab-separated values (TSV) format^2^.

### 2.4 Edge score computation

We developed a lightweight CPU-bound PyTorch neural network to regress a score for each edge in MicrobiomeKG to serve as a centralized semantic unifier, accounting for methodological differences in the underlying knowledge and enabling graph-wide edge interpretations and centrality analyses. To train the model and score edges, we selected 11 features denoting the significance of an edge, the sample size used to make an assertion, whether the significance of an edge was FDR-corrected, the strength of the assertion in an edge, the statistical test used to make an assertion, the type of natural language processing required to compute an edge, the database used to map an edge’s subject and object to a CURIE, and miscellaneous context comprising the notes and supplementary file caption fields. We cast numeric features to a standard normal distribution and label-encoded categorical features. We embedded free-text features with the pooler output of the dmis-lab/biobert-base-cased-v1.1 transformer from HuggingFace (Lee et al., 2020). These features were then passed through three linear layers delimited by LeakyReLU activation functions, with a dropout of 20% between the two largest linear layers to prevent overfitting, given the similarity of certain features. Finally, we leveraged a Softplus activation function after the last linear layer to ensure strictly positive scores. Our specific implementation of the model was trained on 300 manually scored edges, with unique combinations of all 11 features, from initial versions of MicrobiomeKG. During the training loop, we used a Huber Loss (σ = 1) implemented with SmoothL1Loss (β = 1) and an Adam optimizer (Kingma and Jimmy, 2017).

### 2.5 Centrality analysis

We calculated node centralities using four methods from the graph_tool Python library: node betweenness, eigenvector, Katz, and PageRank (Peixoto, 2023). For the Katz centrality method, we set alpha to 80% of the eigenvalue and beta to the eigenvector of the corresponding node. We treated edges with symmetric predicates (biolink:correlated_with and biolink:associated_with) symmetrically, and edges with asymmetric predicates (biolink:affects) directionally. We calculated the edge weights for these analyses using the scoring regression neural network described above.

### 2.6 Deployment

We deployed MicrobiomeKG as a public web application programming interface (API) using Translator Reasoner API (TRAPI) format^3^. We achieve this using Plover (Glen et al., 2025), an in-memory Python-based platform designed to host and serve Biolink-compliant knowledge graphs as TRAPI APIs. Plover enables one-hop queries of the underlying KG and automatically performs Biolink predicate/class hierarchical reasoning and concept subclass transitive chaining, among other tasks. The Plover MicrobiomeKG API is accessible for direct querying via its Translator deployment endpoint^4^.

## 3 Results

### 3.1 Overview of MicrobiomeKG

**FIGURE 1:**
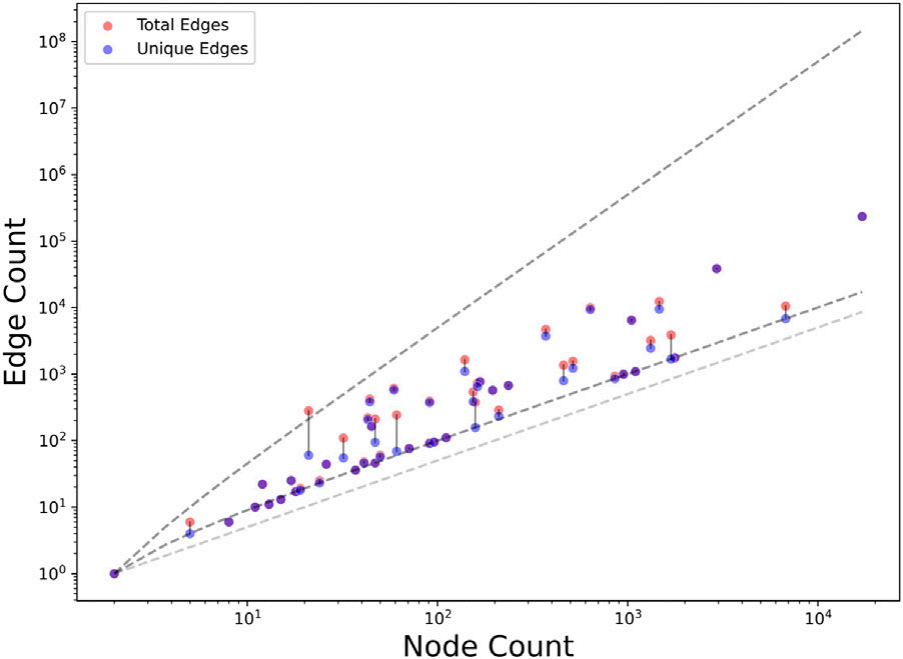
Node, edge, and unique edge count of MicrobiomeKG subgraphs stratified by publication. Each publication is represented by two (potentially overlapping) dots: an orange dot denoting the total number of edges contributed by that publication and a blue dot denoting the number of unique (non-redundant) edges. A gray line connects corresponding dots when they are not next to each other. Dark gray dashed lines denote the theoretical maximum and minimum unique edge counts for a connected graph. These correspond, respectively, to n•(n-1)/2 (for a fully connected graph) and n-1 (for a graph lacking any cliques), where n denotes the number of nodes in the graph. The light gray dashed line denotes the theoretical minimum unique edge count (n/2) without requiring the graph to be connected.

We developed Microbiome KG, a knowledge graph built for microbiome research, focusing on the interface between the microbiome and the health of the host. The current version (2.1.0) contains knowledge assertions crafted from 104 different supplementary tables (Supplementary Table S1) across 40 publications. The number of assertions derived from each publication varies over four orders of magnitude (Figure 1, Edge Count axis), reflecting the huge diversity in content and level of detail of the supplementary tables. The graph components derived from each publication may have multiple separate components, and therefore their unique edge counts may be lower than the expected theoretical minimum for connected graphs (Figure 1, blue dots and lower gray dashed line). Additionally, they may include edges sharing the same subject–predicate–object triple but found in different supplementary tables or using different analytical methods. Their total edge count may therefore exceed the theoretical maximum (Figure 1, orange dots and upper gray dashed line).

**FIGURE 2:**
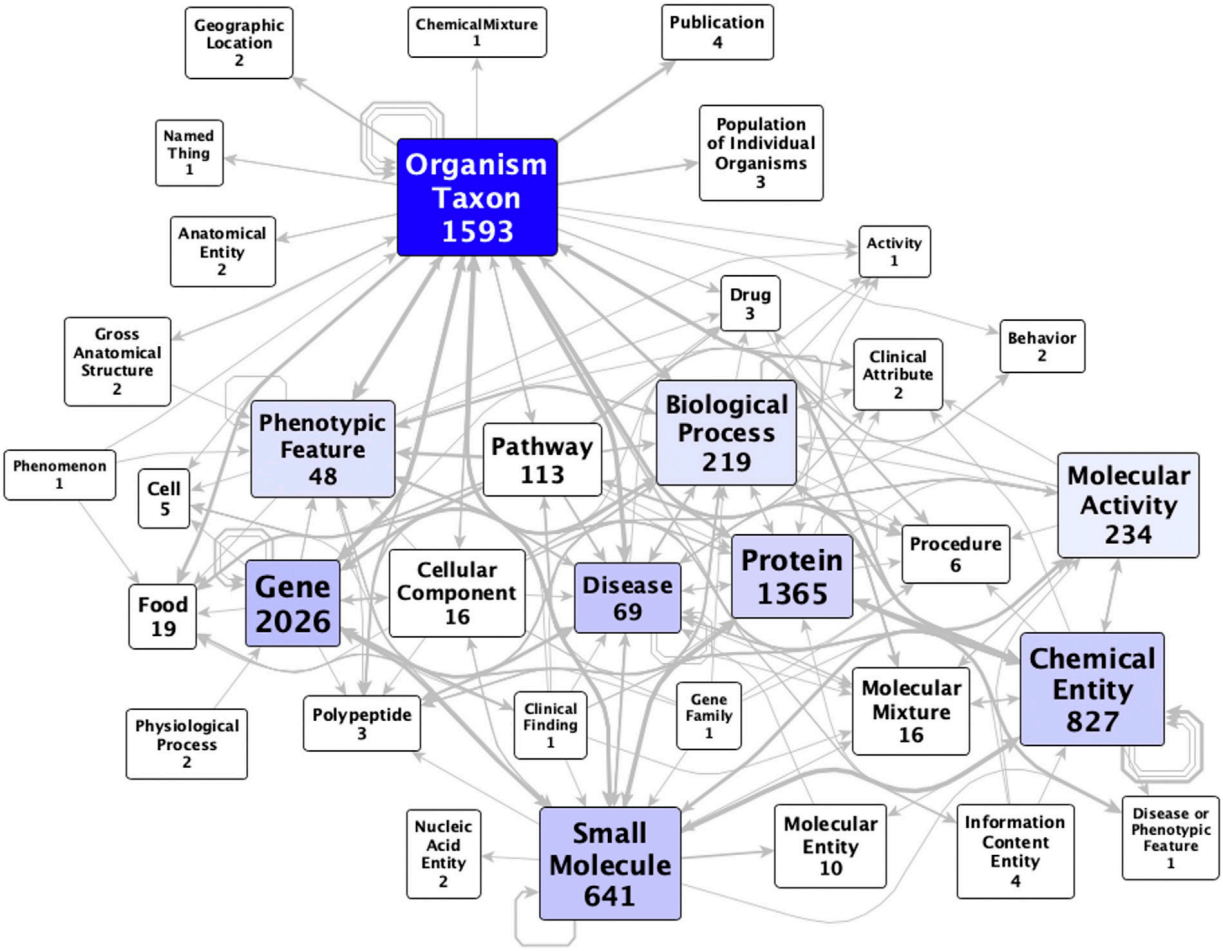
Metagraph of MicrobiomeKG. Vertices represent Biolink ontology classes; numbers in labels represent the count of nodes in the graph that belong to that class. Each arc represents a Biolink ontology predicate (or type of assertion) between two vertices; arcs are designated independent of edge count and are directed. Some pairs of vertices are connected by multiple arcs.

The KG comprises 27,772 nodes (concepts) and 112,118 edges (assertions, of which 71,602 are statistically significant) that outline relationships between the microbiome and various host health factors, spanning 38 Biolink (Unni et al., 2022) ontology classes (most commonly, genes, taxa, proteins, and chemicals—Figure 2; Supplementary Table S2). Disease and SmallMolecule are the most central classes to the graph, followed by OrganismTaxon, PhenotypicFeature, ChemicalEntity, and Gene. Notably, class node count does not correlate to graph centrality. For example, diseases (with 90 nodes) are more central than proteins (with 3,311 nodes), despite a roughly 36-fold ratio in the number of proteins vs. diseases included in the KG (Figure 2). The KG uses eight different biolink ontology predicates, of which the most commonly used are biolink:associated_with and biolink:correlated_with. Taking into account symmetric predicates, there are 244 combinations of subject category, predicate, and object category (Supplementary Table S3), with the most common being “Protein correlated_with SmallMolecule” (22,513 counts).

### 3.2 Case study 1: helminthiasis and NAFLD

**FIGURE 3:**
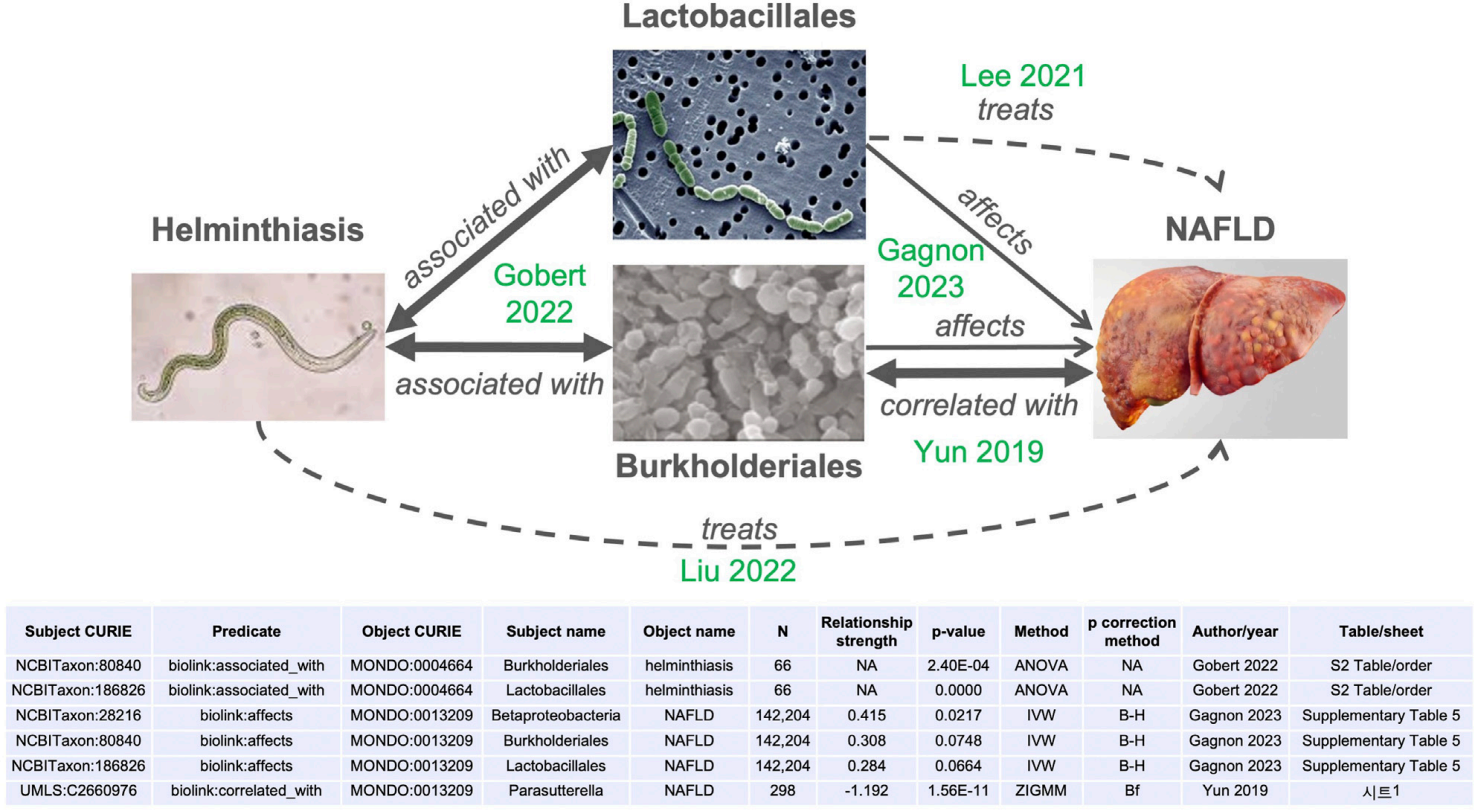
Microbiome effects link helminth infections with non-alcoholic fatty liver disease (NAFLD). Arrows indicate relationships between the concepts (helminth infection, bacterial classes, NAFLD). Bidirectional arrows represent symmetrical relationships (associated with, correlated with). Thicker lines represent significant relationships, dashed arrows represent higher-level “treats” relationships. The source publication for each edge is indicated in green. IVW: inverse variance-weighted. ZIGMM: zero-inflated Gaussian mixture model. B–H: Benjamini–Hochberg. Bf: Bonferroni. NA: not applicable.

Through the combination of edges derived from publications already integrated into MicrobiomeKG (see table in Figure 3), we identified a hypothetical connection between helminthiasis (MONDO:0004664) and metabolic dysfunction-associated steatotic liver disease (also known as non-alcoholic fatty-liver disease, or NAFLD, MONDO:0013209). This connection is consistent with and supported by published observations (Raj et al., 2020; Lee et al., 2021; Liu et al., 2022).

Helminthiasis is a global health burden, particularly in economically underdeveloped regions. Helminth colonization has been linked to changes in host gut microbiomes of increased diversity (Lee et al., 2014). More recent work identified significant alterations in host gut and saliva microbiota, driven by clinical helminth infections (Gobert et al., 2022), at multiple taxonomic levels. Figure 3 highlights the statistically significant association between helminth infections and gut bacteria of the orders Burkholderiales (adjusted p-value = 0.0026) and Lactobacillales (adjusted p-value ∼0), as reported in Supplementary Table S3 of Gobert et al. (2022).

Non-alcoholic fatty liver disease (NAFLD) is a highly prevalent form of progressive and chronic liver disease, with gradual accumulation of liver fibrosis and cirrhosis. The pathogenesis of NAFLD is complex and involves disrupted glycolipid metabolism, inflammation, and dysregulation of the gut microbiota (Han et al., 2023). Metagenomic studies have identified bacterial taxa positively or negatively associated with progression to advanced fibrosis in NAFLD (Loomba et al., 2017). Furthermore, Gagnon et al. (2023) used Mendelian randomization to establish the causal relationships between gut microbiota and multiple cardiometabolic traits and chronic diseases, including NAFLD (Gagnon et al., 2023). We highlight their finding that Class Betaproteobacteria affects (leads to) NAFLD, with a Benjamini–Hochberg adjusted p-value of <0.0218 as computed using the inverse variance weighted (IVW) method (their Supplementary Table S5) and adjusted p-value of <0.000076 calculated using the IVW radial method (their Supplementary Table S6). The association with order Burkholderiales within class Betaproteobacteria did not reach the significance threshold but was suggestive, with an adjusted p-value of <0.075 (their Supplementary Table S5). A significant negative connection between Burkholderiales (specifically, *Parasutterella*) and NAFLD was reported by Yun et al. (2019). Similarly, the relationship with *Lactobacillus* did not reach statistical significance (adjusted p-value <0.0664), but a mechanistic relationship is reported by Lee et al. (2021). Both Burkholderiales and Lactobacillales have potential application as therapeutics for NAFLD (Lee et al., 2021; Liu et al., 2022).

### 3.3 Case study 2: genus *Alistipes* and inflammation

Connections between dietary patterns and systemic inflammation have long been established, with diets that emphasize animal proteins leading to increased inflammation versus diets that emphasize fiber, fruit, and vegetables lowering it (Galland, 2010; Ricker and Haas, 2017). Both diet and inflammation have also been linked to the gut microbiome (David et al., 2014; Zhang et al., 2022; Mirhosseini et al., 2024). In particular, the genus *Alistipes* has been implicated in inflammation (Kaur et al., 2017; Parker et al., 2020), although this assertion is ultimately derived from work that does not support it (Rautio et al., 1997). More recent publications provide additional support for this connection (Wan et al., 2019; Rinninella et al., 2023).

**FIGURE 4:**
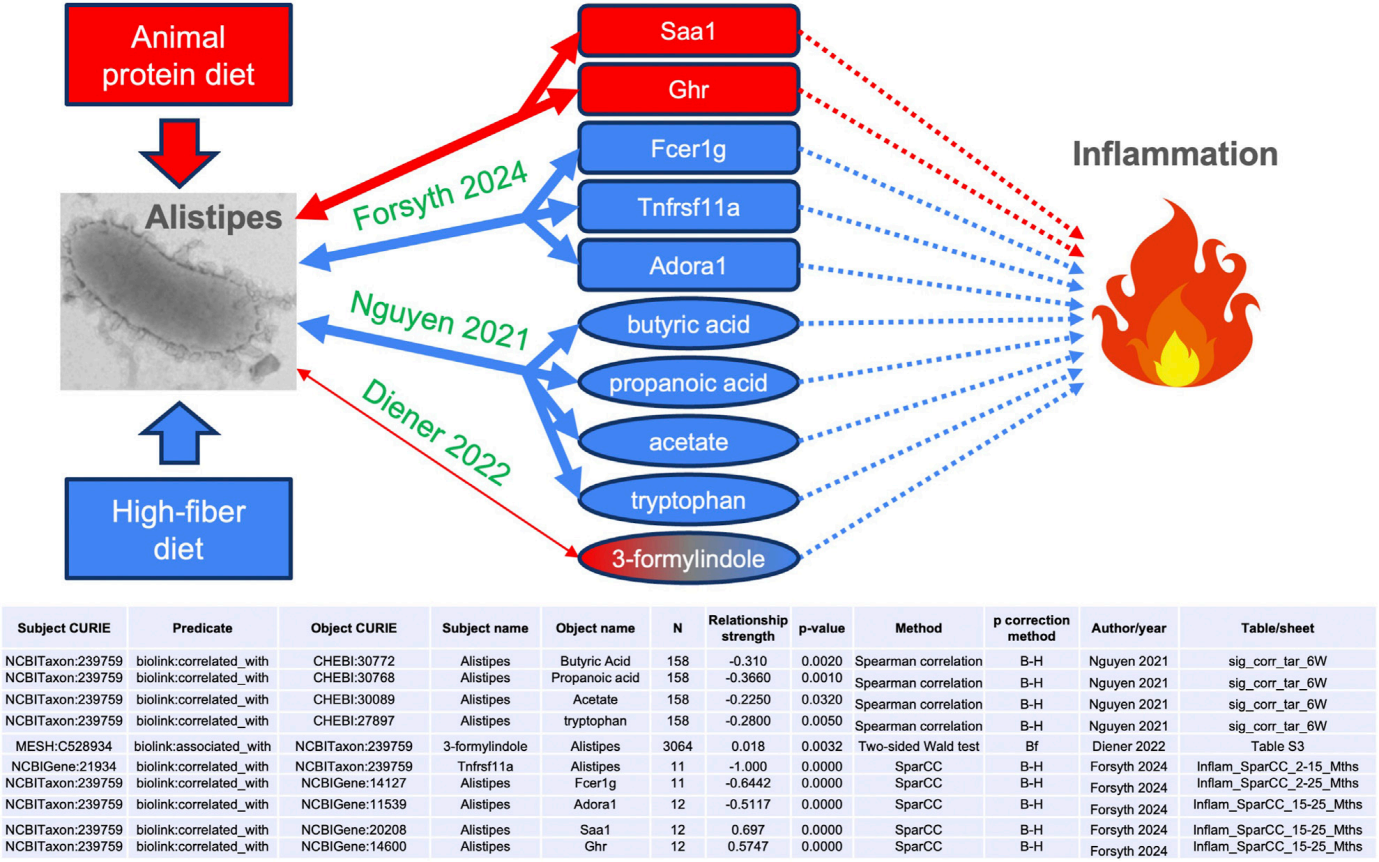
Metabolite and gene connections between the *Alistipes* genus and inflammation. Arrows indicate relationships between the concepts. Bidirectional arrows represent symmetrical relationships (associated with, correlated with). Dotted lines denote multi-step connections, not detailed here. The source publication for each edge is indicated in green. Red and blue denote, respectively, positive (both increase together) and negative (increase in one with decrease in the other) associations. The thinner line for Diener et al. (2022) represents the weaker effect size, and the color gradient for 3-formylindole denotes the inconsistency in directionality. SparCC: sparse correlation for compositional data. B–H: Benjamini–Hochberg. Bf: Bonferroni.

We observed in MicrobiomeKG multiple connections between the genus *Alistipes* and entities associated with inflammation (Figure 4), including genes (*Saa1*, *Ghr*, *Fcer1g*, *Tnfrsf11a*, and *Adora1*), tryptophan-related metabolites (tryptophan and 3-formylindole), and short-chain fatty acids (including butyric acid, propanoic acid, and acetate). The edges supporting these connections are sourced from Forsyth et al. (2024), Nguyen et al. (2021) and Diener et al. (2022).

Nguyen et al. (2021) studied the gut microbial communities and host metabolome in early life (6 weeks and 12 months of age) in humans. While they did not discuss it, their supplementary data show that *Alistipes* was negatively correlated with butyric acid, propanoic acid (which has multiple anti-inflammatory derivative drugs), and acetate (Spearman correlations of −0.31, −0.366, and −0.225, respectively; Benjamini–Hochberg adjusted p-values of 0.002, 0.001, and 0.032, respectively). These three metabolites are short-chain fatty acids (SCFAs) known to have anti-inflammatory effects (Cook and Sellin, 1998; Hamer et al., 2008; Mishiro et al., 2013; Al-Lahham and Rezaee, 2019). The data also indicate a negative correlation with tryptophan (Spearman correlation of −0.28; Benjamini–Hochberg adjusted p-value of 0.005), an essential amino acid that plays a complex role in inflammation, both directly and through its metabolites (Sorgdrager et al., 2019; Seymour et al., 2024; Harris et al., 2024).

Diener et al. (2022) jointly correlated host genetic variants and gut microbiome with the blood metabolome in humans (Diener et al., 2022). Here, the genus *Alistipes* was again not mentioned in the manuscript, but their Supplementary Table S3 reports a weaker positive association with 3-formylindole (two-sided Wald test of 0.018; Bonferroni adjusted p-value of 0.0032). This metabolite is also anti-inflammatory (Luo et al., 2024).

Forsyth et al. (2024) studied the relationship between gut microbiome dysbiosis and inflammaging in mice. They reported that the prevalence of *Alistipes* was positively correlated with the expression levels of the genes *Saa1* and *Ghr* and negatively correlated with *Tnfrsf11a*, *Fcer1g*, and *Adora1* (see table in Figure 4). Serum amyloid A1 (Saa1) is an acute-phase response protein that rapidly increases during inflammation events (Ye and Sun, 2015; Chen et al., 2023). Knock-out of growth hormone receptor (Ghr) in mice leads to reduced inflammation (Masternak and Andrzej, 2012). Dysregulation and ablation of tumor necrosis factor receptor superfamily member 11A (TNFRSF11A) causes autoinflammatory disorders (Jéru et al., 2013; Papatheodorou et al., 2024). Hypomethylation of the Fc epsilon receptor I gamma gene (*FCER1G*), leading to its increased activity, was observed in patients with rheumatoid arthritis compared to control subjects (Podgórska et al., 2022). Similarly, reduced expression of the adenosine A1 receptor (Adora1) led to islet inflammation in a mouse model of Type 1 diabetes (Yip et al., 2013).

In summary, almost all the gene and metabolite associations identified in MicrobiomeKG connecting the *Alistipes* genus to inflammation are consistent with a pattern of increased *Alistipes* fraction correlating with increased inflammation, both by positively correlating with genes and analytes that are themselves positively associated with inflammation or through two negative associations (e.g., *Fcer1g* and butyric acid). The only exception is 3-formylindole, which is positively associated with *Alistipes* but negatively associated with inflammation; we note that the *Alistipes*–3-formylindole association has a very weak effect size, much lower than the other nine *Alistipes* associations discussed here (see table in Figure 4).

### 3.4 Graph standardization via edge scores

By design, MicrobiomeKG is a highly heterogeneous graph. It integrates knowledge from 290 unique analyses comprising many different methods. Thus, the statistics annotating each edge may often convey a variety of meanings. This poses a unique challenge for graph-wide edge interpretation and centrality analyses. Without a centralized semantic unifier, edges are difficult to compare, particularly at scale, hindering downstream analysis. Furthermore, the complex non-linear nature of how each edge’s features contribute to its accuracy and utility precludes reasonable algorithmic construction of such semantic unifiers (e.g., algorithmic quantifications accounting for how methods affect relationship strength are often weak, especially as the number of different methods scales). However, given the ability to quantify an edge’s accuracy and utility on a small scale, neural networks present a unique and highly scalable solution to this centralized semantic unifier problem. We therefore implemented and trained a neural network (see “Methods”) and computed a score for each edge in MicrobiomeKG. The resulting distribution of scores (Supplementary Figure S1) is approximately bimodal, with each mode roughly corresponding to Boolean edge significance. The softplus activation function in the scoring network implies that theoretical scores should be non-negative (i.e., ranging from 0 to positive infinity). The observed scores for 112,118 edges range from 0 to ∼178, with an average score of ∼74 and very few outliers (Supplementary Figure S1). We then used these scores as edge weights to compute centrality metrics (see “Methods”, and case study 3 below).

### 3.5 Case study 3: ADHD and Bifidobacterium

We computed the most central organism taxa directly connected with a collection of diseases. Firstly, we iterated through each disease in MicrobiomeKG, creating a subgraph comprising the disease and its direct organism taxon neighbors. We then compared the various node centralities of the taxon nodes in these subgraphs, selecting the most central node for interpretation. This analysis identified 39 diseases connected to microbial taxa by four different centrality algorithms (Supplementary Table S4) and only taking into account edge directionality, weighted edges, and direct disease–taxon relationships. In some cases, different centrality metrics highlighted different taxa for the same disease, but frequently the same taxon was identified by most or all centrality methods.

In this analysis, the genus *Bifidobacterium* was the most central organism taxon connected to attention-deficit hyperactivity disorder (ADHD) in MicrobiomeKG across all four computed centralities. The relatively high node betweenness centrality of this genus emphasizes its role as the primary bridge connecting the ADHD node to the rest of the graph. Strengthening this narrative, *Bifidobacterium’s* sizable Katz and eigenvector centralities suggest that the node is strongly influential throughout MicrobiomeKG. Furthermore, the taxon is strongly recursively connected, as indicated by its PageRank centrality (Supplementary Table S4).

This central role that *Bifidobacterium* plays in ADHD is also reflected in the current microbiome-ADHD literature. The genus is described as one of the greatest mysteries in the field, with its relative abundance unpredictably fluctuating with age in different populations with ADHD (Cickovski et al., 2023). Furthermore, supplementation with *Bifidobacterium bifidum* (Bf-688) has yielded promising results in reducing inattentive and hyperactivity/impulsivity in clinical trials (Wang et al., 2022; Wang et al., 2024).

## 4 Discussion

We here present MicrobiomeKG, a novel knowledge graph connecting the microbiome and host health, and three case studies highlighting its application. MicrobiomeKG derives knowledge assertions drawn from supplementary materials published together with microbiome papers. Unlike the standard application of natural language processing of paper abstracts and/or full texts of papers, which is perforce limited to content their authors decided to discuss in the text (and, potentially, the main-text tables), content extraction from the supplementary tables may capture a significantly larger corpus of knowledge assertions not included in the manuscript for a variety of reasons, including considerations of statistical significance, space limitations, and decisions about focus of narrative. In some cases, the supplementary tables provide precise numerical values for content included in the manuscript narrative in a simplified or approximate form, or perhaps in graphical form in embedded figures, which pose additional data extraction challenges. By table-mining the supplementary materials, we are thus able to maximize knowledge extraction while minimizing reproduction errors. For example, most of the edges underlying case studies are not in their papers’ main text, tables, or figures, yet they are readily derivable from the supplementary data tables. Supporting materials from publications have been used to extract gene sets (Clarke et al., 2024); here, we applied them to extract structured knowledge assertions. Previous efforts have already extracted knowledge from the full text of published manuscripts via natural language processing or through wholesale inclusion in the training of large language models (LLMs). MicrobiomeKG is designed to supplement (and be integrated with) such existing knowledge bases, not to replace them or be redundant with them.

There is a need in the field for work that validates assertions by comparing results from different datasets and identifying inconsistencies in the assertions reported by different studies, as collected in large repositories like MGnify (Mitchell et al., 2020). A goal of the current project is to facilitate such efforts by collecting and standardizing the representation of such assertions as made available in the supplementary materials of published papers. Even after standardizing the semantic representation of the assertions, the heterogeneity of contexts, methodologies, and reporting standards used in the different studies pose an additional challenge for the integration, comparison, and downstream analysis of the edges in the knowledge graph. We thus developed an approach to scoring edges into a standardized framework. We achieved this by applying neural networks to integrate multiple aspects of publication and edge metadata such as sample size, statistical test and correction methods, and context terms derived from the manuscript itself. We demonstrated the use of such standardized edge scores to compute centrality metrics (Muhiuddin et al., 2023), which we then used to rank hypotheses within MicrobiomeKG subgraphs of interest, such as which organism taxa are directly related to specific diseases.

The resulting KG is available for direct download and is also deployed via Plover (Glen et al., 2025) and integrated with other KGs through the Translator ecosystem, which already incorporates assertions derived from other knowledge bases. Use of the KGX exchange format^6^, Biolink model (Unni et al., 2022) categories and predicates, and the standardized normalization of all terms into CURIEs, ensures the interoperability of the resource. This can be easily transformed into other knowledge representation and exchange formats, like BioRDF (Nolin et al., 2010) and integrated with cross-referenced data from other microbiome resources like MGnify (Mitchell et al., 2020).

A limitation of MicrobiomeKG is its current scope. The version of the graph presented here contains 27,772 nodes and 112,118 edges sourced from a set of 40 microbiome papers (Figure 1). Disbiome, a prior effort that manually curates information linking the microbiome with a disease, included assertions sourced from approximately 500 papers upon publication (Janssens et al., 2018) and then expanded to 1,179 papers—a much larger collection than currently included in MicrobiomeKG. On the other hand, that manual curation effort yielded 10,866 assertions linking 1,615 organisms to 375 diseases, which is a very limited number compared to the node and edge count in MicrobiomeKG. Likewise, the MGnify resource includes over 3,500 publicly available projects connected with 1,785 microbiome publications (Mitchell et al., 2020), although the scope is much wider than the microbiome-to-disease domain. To scale up the scope of MicrobiomeKG, we plan to implement automated extraction methods to further mine supplemental data for assertions on microbiome and host health while simultaneously expanding the types of multiomic analysis and data types to be included in the graph. In the long-term, we plan to leverage a collection of rule-based algorithms, natural language processing, artificial intelligence, and machine learning methods (including large language models) to optimize data collection and scalability and to improve the metadata associated with the knowledge assertions (Nassar et al., 2022).

Supplementary materials can be very difficult to use (Pop and Salzberg, 2015). By identifying knowledge assertions from supplementary tables and expressing them as knowledge graphs, we are casting this valuable content into a format that is ideal for hypothesis generation. MicrobiomeKG ultimately brings novel nodes and edges to Translator that foster previously unexplored connections between the microbiome and varied biomedical data. We expect that MicrobiomeKG will be the first of many knowledge graphs built from knowledge assertions derived from the trove of untapped supplementary tables. In the context of graph machine learning, such extended knowledge extraction will prove advantageous for training microbiome, biological, biomedical, and host health AI/ML models (Tiddi and Schlobach, 2022). As the field evolves, we foresee the integration of more diverse datasets into knowledge graphs, enhancing the richness and applicability of these resources. This expansion will not only strengthen the predictive power of AI/ML models but also enable data-driven insights into the complex interplay between the microbiome and host health. For example, graph embedding could integrate MicrobiomeKG’s expert-derived insights within graph neural networks, capturing microbial relationships and functional associations to enable downstream analyses such as phenotype classification, differential analysis, and microbial network exploration (Ma et al., 2024). Ultimately, by bridging data gaps and facilitating the discovery of new biological relationships, MicrobiomeKG will help advance personalized medicine through a deeper understanding of microbial contributions to human health and disease mechanisms.

## Data Availability

The original contributions presented in the study are included in the article/Supplementary Material; further inquiries can be directed to the corresponding author.

## Footnotes

1
https://github.com/TranslatorSRI/Babel

2
https://github.com/biolink/kgx

3
https://github.com/NCATSTranslator/ReasonerAPI

4
multiomics.transltr.io/mbkp

6
https://github.com/biolink/kgx
