# Correction: MicrobiomeKG: bridging microbiome research and host health through knowledge graphs

**DOI:** 10.3389/fsysb.2025.1710604

**Published:** 2025-10-27

**Authors:** Frontiers Production Office

**Affiliations:** Frontiers Media SA, Lausanne, Switzerland

**Keywords:** systems biology, data integration, supplementary data, table mining, knowledge representation, health informatics, hypothesis generation, neural networks

Affiliation Elsevier ltd., United Kingdom for reviewer Maaly Nassar was erroneously given as SciBite, United Kingdom.

The original article has been updated.

