# Supplementary figures and images for: MicrobiomeKG: bridging microbiome research and host health through knowledge graphs
Source: Front Syst Biol. 2025 Aug 29;5:1544432. doi: 10.3389/fsysb.2025.1544432 (PMC12425944; doi:10.3389/fsysb.2025.1544432)

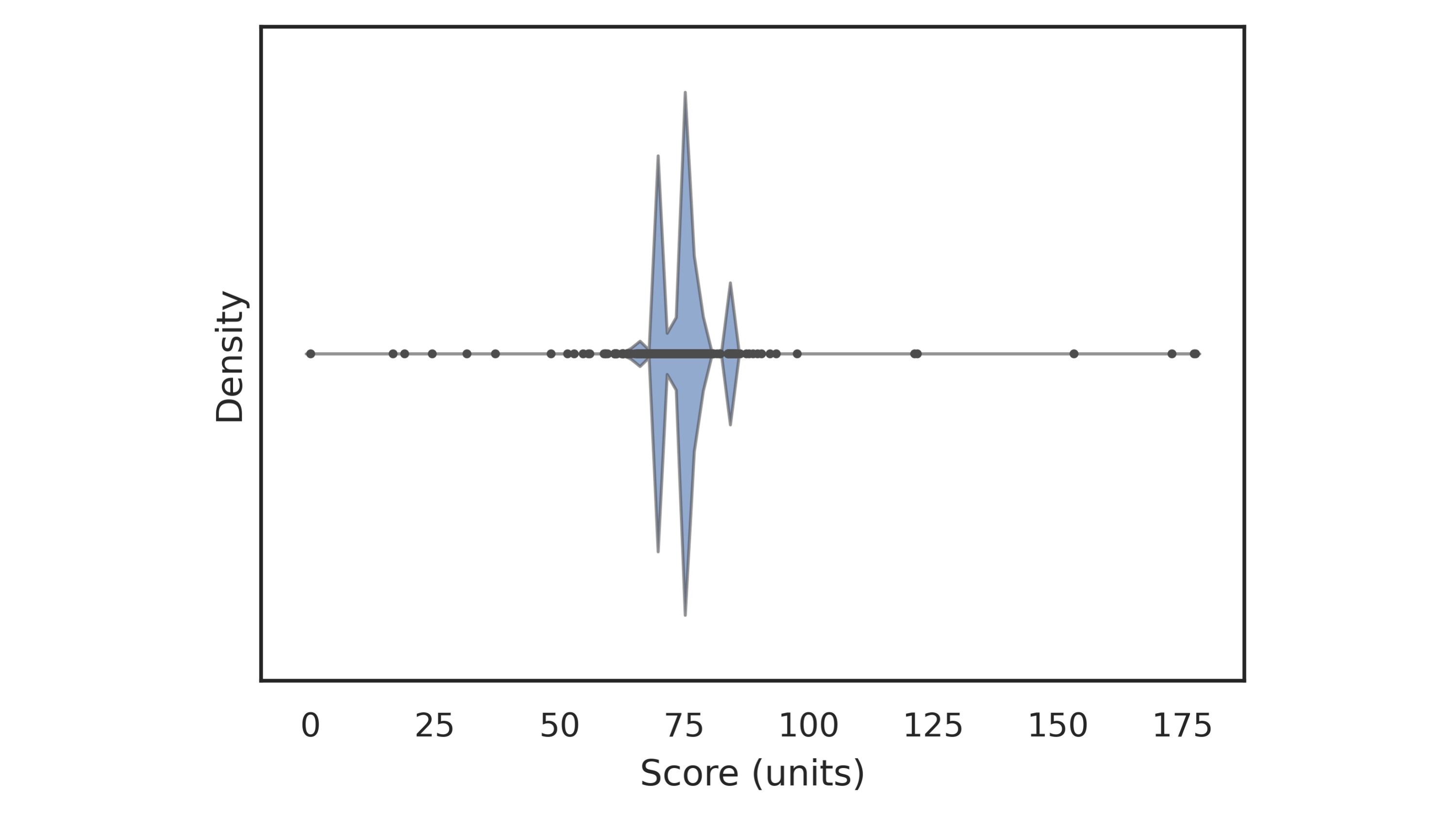

Supplement: Supplementary file 3 [file Image1.jpeg]
